# Combining Noninvasive Brain Stimulation and Physiotherapy to Improve the Management of Chronic Low Back Pain in Veterans: Protocol for a Multi-Arm Randomized Controlled Trial

**DOI:** 10.2196/78952

**Published:** 2026-01-26

**Authors:** Frederique Dupuis, Yannick Tousignant-Laflamme, Pascale Marier Deschênes, Philippe Fournier, Ildephonse Nduwimana, Orlane Ballot, Loris Loisel, Anne Marie Pinard, Luc Lacombe, Pierre Langevin, Alain Gaumond, Jean-Sébastien Roy, Hugo Massé-Alarie

**Affiliations:** 1 Centre for Interdisciplinary Research in Rehabilitation and Social Integration Quebec, QC Canada; 2 École des Sciences de la Réadapation, Faculté de Médecine Université Laval Québec, QC Canada; 3 School of Rehabilitation Université de Sherbrooke Sherbrooke, QC Canada; 4 Centre Hospitalier Universitaire de Sherbrooke Sherbrooke, QC Canada; 5 Chronic Pain Service CHU de Québec-Université Laval Quebec Canada; 6 Canadian Armed Forces Québec, QC Canada; 7 Espace Pleine Conscience Québec, QC Canada

**Keywords:** low back pain, chronic pain, rehabilitation, transcranial magnetic stimulation, psychosocial, physiotherapy, military

## Abstract

**Background:**

Low back pain (LBP) is the most common chronic pain condition in veterans, but the effectiveness of standard management approaches is modest. Addressing the psychological risk factors of chronic pain that are often observed in this population (eg, anxiety, depression, stress and mood disorders) may be important to enhance outcomes. Psychologically informed physiotherapy (PiP) identifies and mitigates the negative impacts of emotional and cognitive factors alongside the biomedical aspects of chronic LBP to improve physical functioning and has shown promising results in this population. However, residual pain and disability often persist in veterans. The combination of PiP with repetitive transcranial magnetic stimulation (rTMS) to the prefrontal cortex may enhance its effectiveness by modulating cognition, emotion, and pain perception.

**Objective:**

The aim of this study is to compare the effects of (1) combining active rTMS with PiP, (2) combining sham rTMS with PiP, and (3) usual physiotherapy (UP) on physical functioning in veterans with chronic LBP and comorbid psychological risk factors. Secondary objectives include comparing their effect on pain intensity, quality of life, depression symptoms, pain catastrophizing, movement pain–related fear, self-efficacy, medication use, and posttraumatic stress disorder symptoms.

**Methods:**

Ninety-six veterans with chronic LBP and comorbid psychological risks factors of pain will be enrolled in this 3-arm parallel randomized controlled trial. Individuals will be allocated to receive an 8-week intervention of (1) active rTMS + PiP, (2) sham rTMS + PiP, or (3) UP. Online self-administered questionnaires will be completed at baseline, 2, 8, and 26 weeks after the first treatment session. A linear mixed model will be used to assess the treatment effects by using intention-to-treat analyses. We hypothesize that active rTMS + PiP will be more effective than sham rTMS + PiP and that active PiP + rTMS or sham rTMS will be more effective than UP.

**Results:**

Ethics approval was obtained in January 2025, and participating physiotherapists completed the 2-day PiP training in May 2025. Participants have been recruited since June 2025. As of December 2025, 28 participants have been included, and recruitment is expected to continue up to June 2027, targeting the inclusion of approximately 4 new participants per month. Follow-up should be completed by December 2027, and results will be analyzed. The results of this randomized controlled trial should be published and available in June 2028.

**Conclusions:**

It is paramount to identify innovative and effective interventions for the management of chronic LBP in veterans. This study will provide new evidence on the effectiveness of two innovative interventions targeting cognitive and emotional factors of pain (ie, PiP and rTMS). If our hypothesis is confirmed, it could motivate changes in clinical practice and improve the quality of life of veterans living with chronic LBP.

**Trial Registration:**

ClinicalTrials.gov NCT06999772; https://clinicaltrials.gov/study/NCT06999772

**International Registered Report Identifier (IRRID):**

PRR1-10.2196/78952

## Introduction

Chronic low back pain (CLBP) stands as the leading cause of years lived with disability globally [1,2]. Addressing this widespread chronic condition poses a notable challenge to both the public and military health care systems. The effectiveness of standard management approaches for CLBP has been found to be modest in both general [3] and military/veteran [4,5] populations, further negatively impacting the quality of life of these people.

In military veterans, CLBP is the most prevalent painful condition [6,7], with prevalence rates reaching 45% [7]. Moreover, veterans are more likely to experience more severe pain intensity and pain-related disability than the civilian population [8]. The poor response to current interventions in this highly impacted population exacerbates the current opioid crisis [9], urging the need for new, effective, and noninvasive interventions.

The limited effectiveness of current interventions suggests that they may not adequately target specific mechanisms underlying CLBP. In the last few years, a paradigm shift has been noted in CLBP management, transitioning from a strictly biomedical perspective to a more comprehensive biopsychosocial approach [10]. Moreover, our understanding of the mechanisms of CLBP has evolved. For example, evidence reveals abnormal brain functioning among individuals with CLBP [11], suggesting the involvement of alterations within the central nervous system in certain people [12]. As the painful symptoms persist, a shift from circuits processing nociceptive inputs to circuits processing emotional states may occur in those at the highest risk to develop chronic pain [13,14]. The corticolimbic system (including the prefrontal cortex [PFC] and limbic system), which is critical in the regulation, processing, and control of emotions, plays a significant role in the inhibition of pain through cognitive control mechanisms within the cortex [14]. Enhanced cognitive and emotional valuation of pain may act as an amplifier of nociception [14]. This transition aligns with the identified multidimensional factors contributing to chronicity [15].

Psychological disorders like depression and anxiety and factors like pain catastrophizing, and movement pain–related fear have repeatedly been linked to the persistence of LBP and long-term disability [16,17]. These psychological factors represent higher-order cognitive and emotional processes occurring, *inter alia*, within the PFC [18], and alterations in brain processing may exacerbate nociception and contribute to the persistence of pain. This physiological phenomenon is referred to as nociplastic pain mechanisms [19]. Considering that psychological factors are more prevalent among veterans compared to civilians [7,20] and are associated with the emergence of chronic pain in this population [7,20-22], there might be a critical need for interventions identifying and mitigating these factors [23]. However, they are frequently overlooked in standard health care practices [24,25].

Usual physiotherapy (UP) constitutes a standard health care intervention for veterans experiencing LBP, and although being effective to improve symptoms and disability compared to no intervention, it often fails to consider the psychological prognostic factors of persistent pain. Psychologically informed practice utilizes a biopsychosocial approach to recognize and address emotional and cognitive components associated with the condition, using different therapeutic modalities such as education, gradual exposure to movement, and stress management strategies [26]. When delivered by physiotherapists combined with UP care, the psychologically informed practice is called psychologically informed physiotherapy (PiP) [26]. PiP represents a promising intervention for the management of CLBP, especially in veterans, as it targets the highly prevalent psychological factors present in this population. This approach is currently supported by evidence from civilian populations: meta-analyses have shown that integrating psychological interventions with UP leads to greater improvements in pain intensity and physical functioning in people with CLBP [27], including those presenting comorbid psychological factors [28], when compared to UP alone.

In addition to PiP, noninvasive brain stimulation over the PFC by using repetitive transcranial magnetic stimulation (rTMS) may be useful to modulate the cognitive and emotional processing involved in pain mechanisms [29,30]. This approach is supported by large randomized controlled trials in psychiatric populations [29]. rTMS depolarizes neurons under the stimulating coil, and modulates brain excitability [31], leading to changes in network connectivity [29] that have been linked to reductions in symptoms such as rumination and negative bias [29,31]. Through potential similar mechanisms, rTMS over the PFC has been shown to reduce pain intensity in cases of neuropathic pain [32], a finding that may relate to the high prevalence of psychological comorbidities among people with chronic pain [16,17]. To date, most studies that have investigated the use of rTMS for chronic musculoskeletal pain stimulated the motor cortex (M1), yielding conflicting results [33-35]. Stimulating the PFC could optimize results by targeting brain areas specifically involved in pain modulation. A pilot randomized controlled trial (RCT) (n=20) studied the efficacy of noninvasive brain stimulation over a prefrontal cortical area in veterans with CLBP, and this stimulation resulted in a large improvement in the physical functioning and depressive symptoms compared to a placebo [36]. PiP and rTMS are two promising interventions specifically targeting psychological factors and cortical mechanisms contributing to CLBP in veterans. Despite these promising pilot results, good quality RCTs investigating the effectiveness of rTMS over the PFC for chronic LBP are still needed.

PiP and rTMS are two promising interventions specifically targeting psychological factors and cortical mechanisms contributing to CLBP in veterans. Combining psychologically informed clinical intervention to rTMS could enhance treatment outcomes, as they could both modulate cognitive and emotional processing involved in pain mechanisms, through different pathways [29]. However, the combination of these two interventions has never been studied in chronic musculoskeletal pain and compared to usual care.

Therefore, the main objective of this study will be to compare the effectiveness of (1) combining PiP + active rTMS, (2) PiP + sham rTMS, and (3) UP alone in improving the physical functioning in veterans experiencing CLBP and comorbid psychological factors. The secondary objectives will be to compare their effectiveness on pain intensity, quality of life, movement pain–related fear, pain catastrophizing, self-efficacy, depressive symptoms, medication use, and posttraumatic stress disorder symptoms. We hypothesize that the combination of active rTMS + PiP will result in a larger effect on primary and secondary outcomes compared to PiP + sham rTMS and UP alone. In addition, we hypothesize that PiP with active rTMS or sham rTMS will be more effective than UP.

## Methods

### Study Design

**Figure 1:**
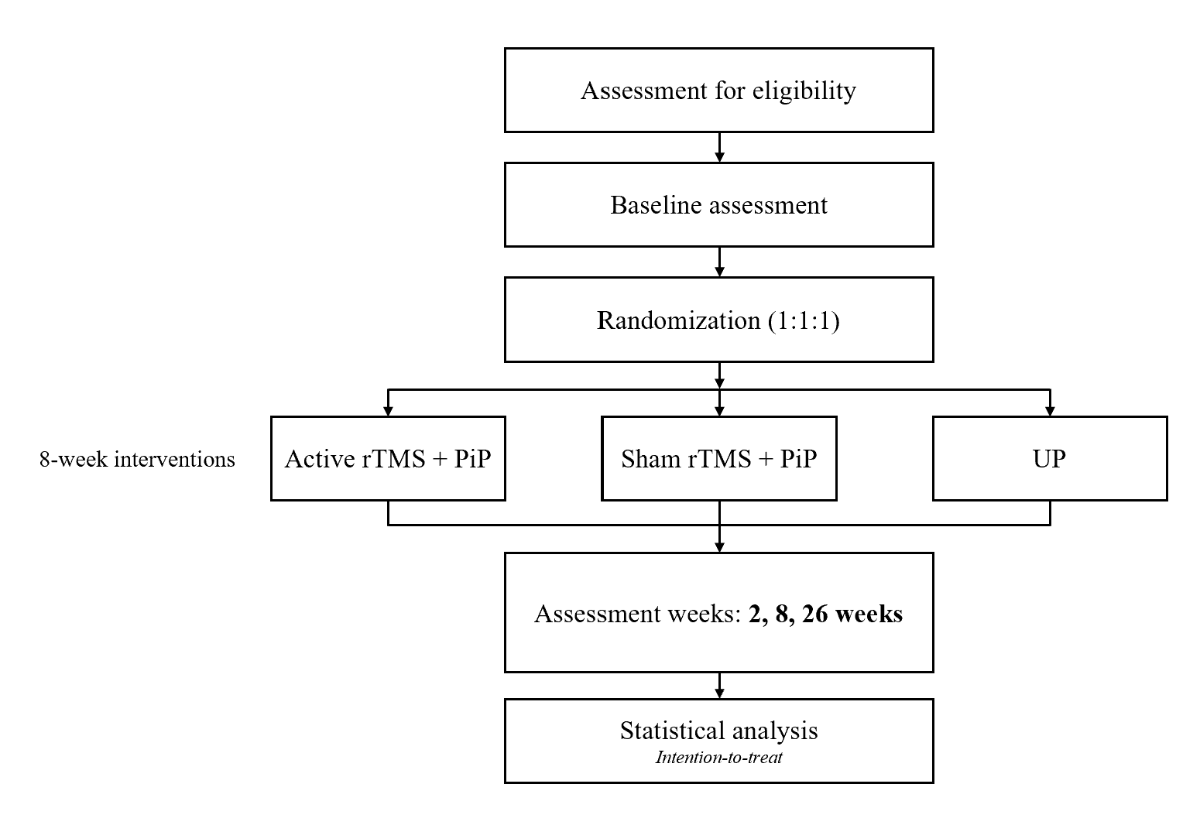
Flowchart of the participants. PiP: psychologically informed physiotherapy; rTMS: repetitive transcranial magnetic stimulation; UP: usual physiotherapy.

This protocol is reported according to the SPIRIT (Standard Protocol Items: Recommendations for Interventional Trials) guidelines [37]. Participants included in this 3-arm, parallel-group RCT will be randomized to one of the 3 groups (PiP + active rTMS, PiP + sham rTMS, or UP) and will undergo an 8-week intervention. Outcomes measurements will occur at baseline, 2, 8, and 26 weeks after the beginning of the intervention (Figure 1). The protocol of this RCT has been registered on ClinicalTrials.gov (NCT06999772).

### Ethical Considerations

Ethics approval has been granted by the Comité d’éthique de la Recherche Sectoriel en Réadaptation et Intégration Sociale du CIUSSS de la Capitale-Nationale (Rehabilitation and Social Integration section; 2025-3215). All participants will provide a written informed consent prior to their participation in the study and will be free to opt out whenever they wish to. Participants will receive compensation of CAD $40 (US $29) after the first 4 weeks of treatment and CAD $40 (US $29) after the last 4 weeks to offset the costs incurred for traveling to appointments. In addition, CAD $20 (US $15) will be given to participants who complete their 6-month follow-up questionnaires in order to increase the retention rate. All collected data using REDCap will be deidentified, and any participant information at recruitment will be kept on an online spreadsheet secured with a password. Only members in the research team have access to this information.

### Study Participants

Veterans with nonspecific CLBP and comorbid psychological factors will be recruited through social media and recruitment emails. A patient partner (LL) will help the team target relevant groups on social media and organizations for the recruitment. Nonspecific CLBP will be defined as LBP that has persisted for at least 3 months and resulted in pain on at least half the days in the past 6 months [38]. A minimal level of disability of ≥15% on the Oswestry Disability Index (ODI) will also ensure that included participants are limited by LBP in their daily activities. The presence of psychological risk factors will be defined as a score of ≥4 on the Star Back Screening Tool [39]. Potential participants will be excluded if they have (1) a change of drug dosage in the last month (ie, drug for the treatment of pain or mental health disorders), (2) any cause of specific LBP (eg, fracture, cancer), (3) any specific rTMS-related exclusion criteria (eg, previous seizure, metal in the head, history of stroke), (4) any confirmed drug or alcohol abuse problem [40], and (5) they cannot understand written French (for self-administrated questionnaires). A research professional or a PhD student will obtain written consent of all the participants before the start of the study after receiving information on the nature and objectives of the study.

### Randomization and Blinding

A randomization list will be generated using a computer random number generator by blocks of 3, 6, or 9 by an independent research assistant. The randomization list will be uploaded on a Research Electronic Data Capture (REDCap, Vanderbilt University) platform by using the randomization module. Once the randomization list will be uploaded, REDCap will automatically retrieve the group allocation. This method digitally replicates the allocation concealment and prevents selection bias without having to manipulate sealed envelopes. Randomization will be stratified by self-reported gender and physical functioning (ODI score ≤ or > 40).

To ensure that no assessor could bias data collection, all patient-reported outcome measures (PROMs) will be assessed using REDCap. Although it is not possible to blind participants to a physical intervention (UP and PiP), they will be blinded to rTMS group allocation (active vs sham). The rTMS intervention will be delivered at the research center, and the physiotherapy interventions will be delivered in local physiotherapy clinics. The 2 interventions (PiP or UP) will be provided in 2 different physiotherapy clinics, reducing the risks of potential group contamination. Furthermore, the physiotherapists providing PiP will not be informed of the type of rTMS (sham vs active), and participants will be asked not to discuss rTMS sessions with their treating physiotherapist. The statistician will be blinded to group allocation. Overall, the RCT will be triple-blind for rTMS (ie, participants, treating physiotherapist, statistician) and single-blind for physiotherapy (ie, statistician). At week 8, participants in the PiP groups will be asked to determine which stimulation they believed they received (sham, active, or don’t know) and to provide the reasons for their choice through a blinding evaluation questionnaire. At any time during the trial, if any severe adverse event occurs (ie, seizure), the research team and the participant will be informed of its group allocation to appropriately manage this event.

### Intervention

**Figure 2:**
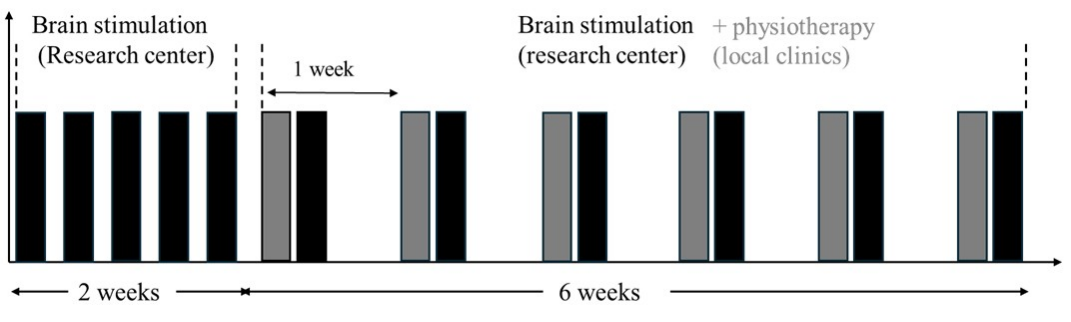
Intervention timelines for the active rTMS + PiP and sham rTMS + PiP groups. Each bar represents a session of intervention. A black bar represents an rTMS (or sham) session offered at the research center, while a grey bar represents a physiotherapy session offered in one of the local clinics. PiP: psychologically informed physiotherapy; rTMS: repetitive transcranial magnetic stimulation.

**Figure 3:**
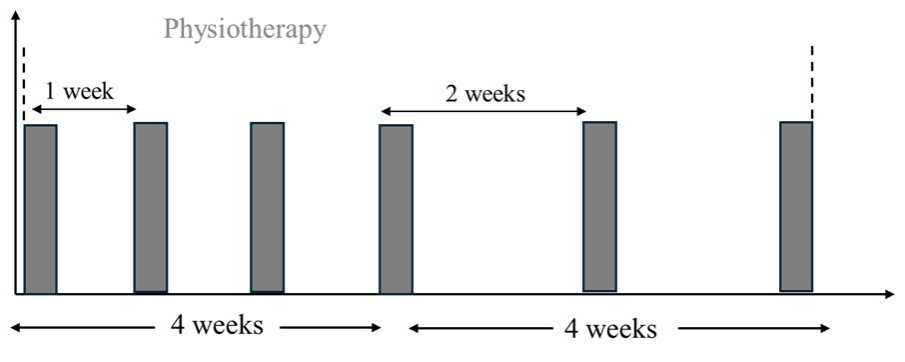
Intervention timelines for the usual care group. Each bar represents a physiotherapy session provided in one of the participating local clinics. In the first 4 weeks, 4 sessions were offered at 1-week interval. In the last 4 weeks of the intervention, 2 additional sessions were offered at 2-weeks interval.

All participants will undergo an 8-week intervention. All participants will receive 6 physiotherapy sessions within 8 weeks, while the rTMS + PiP and sham rTMS + PiP groups will receive 11 additional rTMS sessions in the same period. Figure 2 shows the intervention timeline for the active rTMS + PiP and sham rTMS + PiP groups, while Figure 3 shows the intervention timeline for the UP group. A detailed description of each intervention is provided below.

#### Active rTMS

Participants will receive 11 sessions of rTMS. For the first 2 weeks, participants will attend 5 brain stimulation sessions that will serve as an induction phase to optimize the effects of rTMS [41]. During weeks 3 to 8, 6 rTMS sessions will be provided, once per week. Each rTMS session will last 30-40 minutes (10-20 min of installation + 20 min of stimulation). Stimulation parameters will be obtained by measuring the motor threshold of the right first dorsal interosseous muscle, and then the rTMS intensity will be set at 110% of this motor threshold [32,42]. High-frequency rTMS consisting of 60 trains of 5 seconds at 10 Hz with 15-second intertrain intervals, for a total of 3000 pulses per session, will be used [32]. High-frequency rTMS has been shown to reduce chronic pain [32] and depression symptoms [43]. The coil will be positioned over the left dorsolateral PFC by using the BeamF3 methods [44].

#### Sham rTMS

For the sham stimulation, a sham coil looking like the regular rTMS coil but with a magnetic shield blocking the magnetic field will be used to produce the auditory effects and vibration similar to the active coil. This type of sham has been shown to be effective to blind participants [32]. All other parameters will be the same as the active rTMS.

#### PiP Intervention

The objective of PiP is to identify the biopsychosocial factors that may impede recovery and to address these factors within the physiotherapy scope of practice. To standardize PiP delivery, treating physiotherapists providing PiP will receive a 2-day training provided by an expert in PiP who also has expertise with military personnel. They will learn to use strategies such as the establishment of common goals and therapeutic alliance, the use of the behavior change model, motivational interviewing approach, education on pain neuroscience, gradual exposure to movement, and stress management strategies (eg, breathing techniques, meditation) [45]. The training will also include a section on the military culture to foster therapeutic alliance with veterans and optimize the treatment adherence. It provides foundational knowledge about the Canadian Armed Forces, including its branches, core values, and professional expectations. It supports participants in understanding how military culture influences health behaviors and offers guidance on how this awareness can inform clinical assessments and interventions with veterans. These physiotherapists will also be allowed to use standard physiotherapy modalities presented in the UP section, based on their clinical judgement. However, they will be encouraged to assess and address psychosocial factors by using strategies learned during their training. This training has been shown to be effective in leading to changes in the clinical practice of physiotherapists, leading to a greater use of modalities targeting psychological factors compared to UP [45]. Participants in the PiP + active rTMS and PiP + sham rTMS groups will have access to a website offering support information on topics such as pain neuroscience education, common myths and misconceptions about back pain, the psychological factors influencing pain, self-management strategies, and healthy lifestyle habits (eg, sleep and nutrition). This site will be freely available to participants during the study. Physiotherapists will be asked to discuss one topic at each session with the participants to assess and reframe (if necessary) their conceptualization of these concepts.

#### UP Intervention

This pragmatic group aims to represent real-world clinical practice, including interventions commonly used in physiotherapy to manage CLBP. No training will be provided, and all modalities typically used by physiotherapists in the province of Quebec will be allowed, including but not limited to manual therapy, active exercises, education, stretching, etc. Each physiotherapy session, including PiP and UP, will last between 30 and 45 minutes. Physiotherapists providing UP and PiP will all complete a checklist to indicate the interventions provided at each treatment session.

### Outcomes

#### Overview

**Figure 4:**
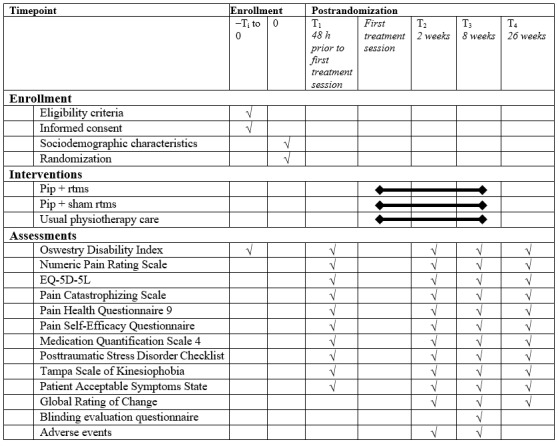
SPIRIT (Standard Protocol Items: Recommendations for Interventional Trials) diagram. PiP: psychologically informed physiotherapy; rTMS: repetitive transcranial magnetic stimulation.

All PROMs will be assessed and filled online by using REDCap. Sociodemographic information will first be collected before randomization. PROMs baseline measurements will be measured after randomization, just prior to the beginning of the treatment. This decision was taken because the time before care initiation can vary depending on the groups (rTMS vs no rTMS) and the clinics delivering the different physiotherapy approaches (PiP vs UP). A longer delay before care initiation for a specific group may influence the outcomes trajectories. The baseline primary outcome will be measured 48 hours prior to the beginning of treatment. Nonetheless, the primary outcome will also be collected before randomization (during enrollment for screening purpose) to ensure similar between-group evolution until baseline measurement. However, participants will be informed on their allocated groups only after baseline PROMs to avoid any anticipation bias. PROMs will then be measured again via email to participants at 2, 8, and 26 weeks after the first treatment session. Figure 4 presents the SPIRIT diagram of this study.

#### Primary Outcomes

The primary outcome will be physical functioning, which will be evaluated using ODI, a 10-item questionnaire (0=no disability; 100=extremely severe disability) that is valid and reliable and recommended to evaluate physical functioning in LBP [46]. The primary time point will be at 8 weeks.

#### Secondary Outcomes

The average pain intensity over the last week will be assessed with the 11-point numerical pain rating scale (0: “no pain” to 10: “worst pain you can imagine”). The numerical pain rating scale is reliable and responsive to change and recommended in LBP trials [46]. The quality of life will be measured using the EQ-5D-5L, which is a generic questionnaire evaluating five dimensions of health-related quality of life, each rated on a 5-point scale [47]. Depression symptoms will be measured using the Patient Health Questionnaire (9 items), which is reliable and valid [48]. Pain catastrophizing will be measured using the valid and reliable Pain Catastrophizing Scale [49]. Movement pain–related fear will be assessed with the Tampa Scale of Kinesiophobia questionnaire (13 items), which has good reliability and validity [50]. Self-efficacy will be measured using the Pain Self-Efficacy Questionnaire, which is valid, reliable, and responsive to change in a population with CLBP [51]. Medication use will be measured using the Medication Quantification Scale Version IV that quantifies 22 drug classes used in pain management (eg, opioid) with a detriment weight [52]. Posttraumatic stress disorder symptoms will be assessed with the Posttraumatic Stress Disorder Checklist for Diagnostic and Statistical Manual of Mental Disorders, which is a 20-item self-report questionnaire that is consistent and valid [53]. The Patient Acceptable Symptom State will be used to determine if the participants consider their current functional and symptom states as satisfactory or not. In addition, the Global Rating of Change, an 11-point scale ranging from –5 (a great deal worst) to 5 (a great deal better) will be used to measure the perceived change of health status after the intervention (ie, at 8 weeks and 26 weeks). Finally, all adverse events and reasons for drop-out will be recorded and reported.

#### Analysis

##### Sample Size Calculation

The trial is designed to detect a 9-point (SD 10) between-group difference in physical functioning at 8 weeks, as measured using the minimal important difference of the ODI [54]. The standard deviation was estimated from our previous RCTs [45,55]. Considering 20% loss at follow-up, 1-β=.90, and α=.05, 32 participants per group will be recruited (n_total_=96).

#### Statistical Analysis

A linear mixed model will be used to compare treatment effects on outcomes, using intention-to-treat analyses. Group (active rTMS + PiP, sham rTMS + PiP, and UP) and time (baseline, 2, 8, 26 weeks) will be entered as fixed factors, participants as random intercept, and stratification factors (gender and baseline ODI score) as covariate, to determine the presence of any significant group × time interaction. Missing values will be handled with multiple imputations if necessary, and sensitivity analyses will be completed to confirm/infirm the results.

#### Trial Management

The study will be coordinated by the principal investigator. A research professional will be the head coordinator for this project. He and a PhD student will be responsible for the day-to-day management of the RCT by overseeing the recruitment efficacy (eg, rate of recruitment), regularly contacting the participating physiotherapists, and preparing and managing the data collection in REDCap.

## Results

Ethics approval was obtained in January 2025, and participating physiotherapists completed the 2-day PiP training in May 2025. Participants have been recruited since June 2025. As of December 2025, 28 participants have been included, and recruitment is expected to continue up to June 2027, targeting the inclusion of approximately 4 new participants per month. Follow-up should be completed by December 2027, and results will be analyzed. The results of this randomized controlled trial should be published and available in June 2028.

## Discussion

This study will investigate the effectiveness of combining psychologically informed physiotherapy and rTMS over the dorsolateral PFC for veterans with CLBP. The management of veterans with CLBP is challenging. Although LBP is the most prevalent source of chronic pain, only modest therapeutic effects are observed [4,5]. Furthermore, veterans with CLBP experience more severe pain and limitations than civilians and are more likely to be prescribed opioids, increasing the risk of chronic opioid use, overdose, and death [9,56]. Identifying innovative, effective, and noninvasive interventions for the management of CLBP in this population is paramount.

The presence of psychological factors such as anxiety, depression, and stress disorders has been frequently observed in veterans living with chronic pain, and growing evidence suggest that these factors contribute to the persistence and intensity of symptoms [7,20-22]. Therefore, targeting the emotional and cognitive factors of pain with PiP and rTMS interventions might be a solution to further improve the quality of life of these people.

Evidence for the effectiveness of rTMS over PFC in chronic musculoskeletal pain is sparse [57], with previous studies mostly targeting M1 and yielding conflicting results [34,35]. Targeting the dorsolateral PFC could be more effective than M1, as the left dorsolateral PFC is involved in cognition and emotion processing that may negatively influence recovery in patients with chronic pain [31,39]. This is also supported by previous studies yielding promising results on the effectiveness of rTMS over the left dorsolateral PFC for neuropathic pain [41].

Various types of psychotherapy (eg, cognitive behavioral therapy, acceptance and commitment therapy, mindfulness-based approaches), when combined with physiotherapy, have been associated with greater improvements in pain intensity and physical functioning among individuals with CLBP, compared to usual care [27]. Moreover, psychologically informed interventions have shown particularly promising results in the treatment of CLBP among patients presenting comorbid psychological factors [28]. Thus, combining these two approaches could enhance treatment response in veterans with CLBP, a population with a high prevalence of psychological disorders. One possible explanation would be that they both target cognitive and emotional mechanisms involved in pain processing but through different pathways [29]. The hypothesis for an enhanced response when combining the two approaches is supported by previous research conducted in patients with major depression. For example, the combination of cognitive behavioral therapy and rTMS treatment yields greater improvements in symptoms and remission rates than previous RCTs evaluating these treatment modalities alone [29].

This study will address some of the top priorities in the management of LBP, that is, to prioritize noninvasive and nonpharmacological biopsychosocial intervention, as recommended by both civilian [58,59] and veteran [60] guidelines. More specifically, it will provide new evidence on the effectiveness of two innovative interventions little studied to date in veterans with CLBP (ie, PiP and rTMS). Furthermore, while the body of evidence supporting the efficacy of rTMS in treating mental health disorders such as major depression or posttraumatic stress disorders is steadily growing [61], important gaps remain in our understanding of its application among patients experiencing chronic musculoskeletal pain, particularly those presenting psychological risk factors. Should our hypotheses be confirmed and PiP demonstrates superior outcomes compared to UP, the findings of this study could yield substantive clinical implications. For example, such results could motivate clinicians to acquire training in administering PiP, as this training can be easily adapted across various health care professions.

Some limitations should, however, be mentioned. First, the optimal parameters for rTMS treatment over the PFC are still unknown. The parameters for this study are based on available evidence, which has mainly been conducted in patients with neuropathic pain [62-64]. It is possible that they are not optimal for the treatment of CLBP. Second, based on evidence from the population with depression, the use of psychotropic medication may attenuate the treatment response to rTMS [65]. We anticipate a high prevalence of psychotropic medication use among our participants, given the elevated rates of psychological disorders observed in veteran populations [7,20]. Finally, several clinicians will be providing care for whom PiP is a new concept. As such, it is possible that the standardization of the PiP intervention may be suboptimal despite the two days of training provided. To optimize standardization and application of the concepts, clinicians will be able to contact the expert clinician who provided the training throughout the study to validate information about the intervention provided. In addition, clinicians’ logbooks will allow us to see which interventions were used with participants.

## Appendix

Multimedia Appendix 1 Peer review report from the Chronic Pain Centre of Excellence for Canadian Veterans (CPCoE).

## Abbreviations

CLBP: chronic low back pain
LBP: low back pain
ODI: Oswestry Disability Index
PFC: prefrontal cortex
PiP: psychologically informed physiotherapy
PROM: patient-reported outcome measure
RCT: randomized controlled trial
REDCap: Research Electronic Data Capture
rTMS: repetitive transcranial magnetic stimulation
SPIRIT: Standard Protocol Items: Recommendations for Interventional Trials
UP: usual physiotherapy
