# Supplementary material for: Combining Noninvasive Brain Stimulation and Physiotherapy to Improve the Management of Chronic Low Back Pain in Veterans: Protocol for a Multi-Arm Randomized Controlled Trial
Source: JMIR Res Protoc. 2026 Jan 26;15:e78952. doi: 10.2196/78952 (PMC12887568; doi:10.2196/78952)
Supplement: Multimedia Appendix 1 [file resprot_v15i1e78952_app1.pdf]

Scientific Advisory Board Feedback

**Principal Applicant**	Hugo Massé-Alarie
**Institution**	Cirris, CIUSSS Capitale-Nationale
**Funding Opportunity**	Assessing and improving quality of life of Canadian Veterans living with chronic pain and common comorbidities
**Project Title**	Enhancing the effects of psychologically-informed physiotherapy using non-invasive brain stimulation to improve physical functioning in Canadian Veterans living with chronic lower back pain

REVIEWER 1

Relevance

COMMENTS:
The research is highly relevant to the Funding Opportunity since it proposes to measure the effect of brain stimulation to an approach already taken into account the physical and psychological dimensions of chronic pain.

Innovation (Adapted from NIH)

COMMENTS:
This project is not very innovative as it combines already existing and studied interventions.

Background and Rationale

COMMENTS:
Sound and clear.

Objectives

COMMENTS:

Goals and objectives clear, well-defined, and aligned with the expected outcomes.

Methodology

COMMENTS:
The methodology is clear, complete and explicit.

Sex & Gender-Based Analysis Plus (SGBA+)

COMMENTS:
The main objective of the proposal is not to analyse SGBA+, but it is mentioned that the results could give a preliminary idea of the difference in gender if there is one.

Research Team

COMMENTS:
Strong expertise and clear mention of the roles of the different members.

Veteran Engagement

COMMENTS:
A clear and meaningful involvement of veterans is described. Authors even explained how co-construction of the project already lead to modification proposed by veterans.

Feasibility

COMMENTS:
The team clearly identified how to reach the objectives and demonstrated they have the skills, knowledge and experience to complete the study.

Knowledge Mobilization

COMMENTS:
Well described, diverse populations targeted, and different KM products proposed.

Likely Overall Impact

COMMENTS:

This project has a high potential for concrete outcomes for Veterans and will allow to understand the impact of a combination of existing intervention to reduce chronic pain and the related comorbidities.

SUMMARY COMMENTS:

Very good project, well written, strong expertise and experience in this kind of project. The expected outcomes have a strong potential to benefit veterans in the short-term.

REVIEWER 2

Relevance

COMMENTS:

This project is relevant to the funding call.

Innovation (Adapted from NIH)

COMMENTS:

Use of rTMS in psychologically informed physiotherapy (PIP) for back pain is a novel approach to a widespread healthcare issue which may have considerable clinical implications for pain management in future.

Background and Rationale

COMMENTS:

Background is well argued. Exploration of gender/SGBA issues could be discussed in the background.

Objectives

COMMENTS:

Hypotheses are clearly explained and relevant.

Methodology

COMMENTS:

Would be beneficial to include health economics measures (e.g. days off work, contact with hospital/general practitioners/ etc.) in this study to understand the impact of the rTMS and PIP.

A strength of this study is the use of RTMS + PIP, sham rTMS + PIP and usual practice groups.

Sample size is well considered and seems feasible, but it may be useful to consider a contingency plan should n=96 patients prove challenging.

Sex & Gender-Based Analysis Plus (SGBA+)

COMMENTS:

Unclear beyond training what SGBA considerations have been made. Consideration of SGBA in sampling not apparent.

Research Team

COMMENTS:

Research team appear to have necessary skills, equipment and experience to conduct this study.

Veteran Engagement

COMMENTS:

It is a strength of this study that Luc Lacomb is a veteran collaborator. However, more could be done to include a wider range of veterans with different lived experience of back pain to inform the design and dissemination strategy to maximise this study's impact – for example the inclusion of a patient and public involvement group.

Feasibility

COMMENTS:

This project does appear feasible. Contingency plan in case recruitment difficulties are experienced not present. Discussion of possible risk of adverse events of rTMS not discussed.

Knowledge Mobilization

COMMENTS:

Knowledge mobilization plan seems diverse (e.g. videos, lunches, publications). Greater consideration for how the findings could be delivered to veterans and their families could be included as KM appears quite clinician focused at present.

Likely Overall Impact

COMMENTS:

This study could have considerable benefits for veterans living with chronic back pain. However, how veterans will be involved in the study design, analysis and dissemination strategy could be more clearly defined to maximize impact.

SUMMARY COMMENTS:

N/A
